# The Preliminary Training School for Nurses: III. The Week's Work


**Published:** 1917-06-09

**Authors:** 


### THE MATRONS' AND SISTERS' DEPARTMENT.

### THE PRELIMINARY TRAINING SCHOOL FOR NURSES.*

### III. The Week's Work.

On Monday morning probationers are called at 6.15 A.M. and breakfast is at 7 A.M., after this there are prayers, which it is not compulsory to attend; they then do the same housework they did the previous day. This is finished at 8.30 A.M., and they then prepare to go to the lecture-rooms behind the hospital, where they have a physiology lecture from 9.30 to 10.30 A.M., then a light lunch of milk and biscuits, after this a cookery demonstration lasting two hours; returning by 1.30 P.M. to middle-day dinner at the Preliminary Training School.

In the afternoon they are off duty for two hours. Tea is at 4 P.M., and after this various classes of one hour's duration, the probationers being arranged in three divisions so that three classes are carried on at the same time, each probationer attending two classes of one hour each. The time between these classes and supper, which is at 8.30 P.M., is spent in the preparation of lectures, etc. After supper there are short prayers, and then the probationers can either amuse themselves in their sitting-room or go to bed, whichever they prefer, but all are expected to be in their rooms by 10 P.M.

The work is more or less carried on in this way during the week, but on Tuesdays and Fridays the "off-duty" time is two and a half hours and on Saturdays three and a half hours; also on Tuesday and Wednesday the probationers go up to the hospital sick cookery class-room and themselves do the cookery that they have previously seen done at the demonstration. This method is followed in all the subjects, the demonstrations and theoretical work in every case being followed by practical instruction, while the pupil probationers do all the work under trained supervision.

A great point is made of punctuality at meals and classes, and "working to time" is taught after the first three weeks, a definite time being allowed for each thing, whether making a bed, putting on a bandage, or applying a fomentation. In England a few weeks' preliminary training give good results. So long as the number is not too great to allow of personal individual attention to probationers, it seems a pity to keep them waiting longer before the start in the wards.

As salary cannot commence until the actual hospital training is started, many desirable candidates could not afford more than two months without salary, in addition to the initial expenses, without sometimes serious inconvenience to themselves and their parents, even though board, lodging, and washing were provided free. This might stand in the way of those who might otherwise make good nurses for sick people, as in starting a new work it seems important to have the mind, as far as possible, free of other worries; it is not always the most suitable candidates who have the greatest worldly possessions. Of course, it is also very necessary to look at the expenses from the hospital point of view. Those who mean to devote their lives to public service can feel quite justified in being trained at the expense of the hospital, as they can then, by their work, pay it back.

Paying probationers, of whom some are taken for a three months' course—half at the Preliminary Training Home and half at the hospital—pay fifteen guineas, and this more or less covers the cost of their training and food. The routine is exactly the same for them as for the non-paying probationers, but they can then feel free to leave at any time by mutual arrangement. From those who do not wish to pay for training and take up nursing without sufficient thought or "just to see how they like it," or from curiosity, it is necessary to protect both the hospital funds and those who are devoting their time to training probationers, so a sum of £10 has to be paid if a probationer leaves of her own accord during her time at the Preliminary Training School; this prevents this individual experiment being a dead loss to the hospital. Another advantage is that it acts as a check on an impulsive woman, and makes her think before deciding to "throw it all up," because for the moment she happens to feel disheartened, and does not find exactly what she expected. Some of those who start in this manner and feel inclined to return home, if they persevere, make excellent nurses eventually.

### ROUND THE HOSPITALS.

* Previous articles appeared May 26, p. 155, and June 2, p. 175.

